# Clubroot resistance gene *Rcr6* in *Brassica nigra* resides in a genomic region homologous to chromosome A08 in *B. rapa*

**DOI:** 10.1186/s12870-019-1844-5

**Published:** 2019-05-29

**Authors:** Adrian Chang, Mebarek Lamara, Yangdou Wei, Hao Hu, Isobel A. P. Parkin, Bruce D. Gossen, Gary Peng, Fengqun Yu

**Affiliations:** 1 Agriculture and Agri-Food Canada, Saskatoon Research and Development Centre, Saskatoon, SK S7N 0X2 Canada; 2 Department of Biology, University of Saskatchewan, Saskatoon, SK S7N 5E2 Canada

**Keywords:** *Brassica nigra*, *Brassica rapa*, Clubroot, *Plasmodiophora brassicae*, Bulked segregant RNA-sequencing

## Abstract

**Background:**

Clubroot, caused by *Plasmodiophora brassicae* Woronin, is a very important disease of *Brassica* species. Management of clubroot relies heavily on genetic resistance. In a cross of *Brassica nigra* lines PI 219576 (highly resistant, R) × CR2748 (highly susceptible, S) to clubroot, all F_1_ plants were resistant to clubroot. There was a 1:1 ratio of R:S in the BC_1_ and 3R:1S in the F_2_, which indicated that a single dominant gene controlled clubroot resistance in PI 219576. This gene was designated *Rcr6*. Mapping of *Rcr6* was performed using genome sequencing information from A-genome of *B. rapa* and B-genome of *B. nigra* though bulked segregant RNA sequencing (BSR-Seq) and further mapping with Kompetitive Allele Specific PCR (KASP) analysis.

**Results:**

Reads of R and S bulks from BSR-Seq were initially aligned onto *B. rapa* (A-genome; *B. nigra* has the B-genome) where *Rcr6* was associated with chromosome A08. KASP analysis showed that *Rcr6* was flanked by SNP markers homologous to the region of 14.8–15.4 Mb of chromosome A08. There were 190 genes annotated in this region, with five genes (*Bra010552*, *Bra010588*, *Bra010589*, *Bra010590* and *Bra010663*) identified as encoding the toll-interleukin-1 receptor / nucleotide-binding site / leucine-rich-repeat (TIR-NBS-LRR; TNL) class of proteins. The reads from BSR-Seq were then aligned into a draft B-genome of *B. nigra*, where *Rcr6* was mapped on chromosome B3. KASP analysis indicated that *Rcr6* was located on chromosome B3 in a 0.5 Mb region from 6.1–6.6 Mb. Only one TNL gene homologous to the *B. rapa* gene *Bra010663* was identified in the target region. This gene is a likely candidate for *Rcr6*. Subsequent analysis of the *Rcr6* equivalent region based on a published *B. nigra* genome was performed. This gene is located into chromosome B7 of the published B-genome, homologous to *BniB015819*.

**Conclusion:**

*Rcr6* was the first gene identified and mapped in the B-genome of *Brassica* species. It resides in a genomic region homologous to chromosome A08 of A-genome. Based on this finding, it could possibly integrate into A08 of *B. napus* using marker assisted selection with SNP markers tightly linked to *Rcr6* developed in this study.

**Electronic supplementary material:**

The online version of this article (10.1186/s12870-019-1844-5) contains supplementary material, which is available to authorized users.

## Background

*Brassica* species are important oilseed and vegetable crops worldwide. The genomic relationships among these important crop species (diploid and amphidiploid) have been summarized in the ‘Triangle of U’ [1]. The diploid species are *B. rapa* (L.) (AA; *n* = 10), *B. nigra* (L.) (genome BB; *n* = 8), and *B. oleracea* (L.) (CC; *n* = 9) and the amphidiploid species are *B. juncea* (L.) (AABB; *n* = 18), *B. napus* (L.) (AACC; *n* = 19) and *B. carinata* (L.) (BBCC; *n* = 17). The amphidiploid species were derived from interspecific hybridization between the corresponding pairs of the diploid species.

Species containing the B-genome are a useful source of genes when breeding for biotic and abiotic stress tolerance, disease resistance and oil quality [2, 3]. In Canada, transferring disease resistance from *B. nigra* to canola (*B. napus*) has potential to expand the genetic base in canola germplasm [4].

Clubroot, caused by *Plasmodiophora brassicae* Woronin, attacks a wide range of *Brassica* species. In western Canada, it was discovered in canola fields for the first time in 2003, but has spread rapidly [5] and currently poses a serious threat to canola production in Canada. Genetic resistance is the most widely used method for clubroot management. However, resistant sources for canola are limited, and no resistance is available in mustard species (*B. juncea* and *B. carinata*). Interestingly, lines effectively resistant to a broad range of pathotypes of *P. brassicae* have been identified in ancestral diploid species [6, 7], which could greatly broaden the genetic base of clubroot resistance (CR) in *B. napus*, *B. juncea* and *B. carinata*.

Several CR genes have been identified and mapped in *B. rapa*, *B. oleracea* and *B. napus*. For example, more than 10 loci on five chromosomes have been identified in the A-genome of *B. rapa*; *Crr1, Crr2, Crr4,* and *CRc* mapped to chromosomes A08, A01, A06, and A02, respectively [8, 9], and *CRa, CRb, CRk, CRb*^*kato*^, *Rcr1* and *Rcr2* were mapped onto linkage group A03 [8–19]. *CRa*, *CRb*^*kato*^ and *Crr1*, which have been cloned from *B. rapa* [20–22], encode toll-interleukin-1 receptor / nucleotide binding site / leucine-rich repeat (TIR-NBS-LRR or TNL) proteins. In recent studies, six QTLs residing in five CR QTL regions of chromosomes A01, A03, and A08 were identified in *B. rapa* [23] and three strong QTLs (*Rcr4*, *Rcr8* and *Rcr9*) were identified in chromosomes A03, A02 and A08, respectively [24].

In the C-genome of *B. oleracea,* one major resistance gene, *Rcr7* originating from *B. rapa* [25], and over 20 QTLs [26, 27] have been identified*.* So far, at least 20 QTLs involving CR have been identified in the A and C-genomes of *B. napus*. A major gene, *Pb-Bn1*, which confers resistance to two *P. brassicae* collections, has been mapped to chromosome A03, and a minor QTL for each collection was mapped to linkage groups C2 and C9 [28]. Also, 19 QTLs involving race-specific resistance were mapped on eight chromosomes: A02, A03, A08, A09, C3, C5, C6 and C9 [29]. From these 19 QTLs, four were closely linked on A03 and three were linked on A08. However, mapping and identification of CR genes has not been carried out in *Brassica* species containing the B-genome.

Next-generation sequencing (NGS) has been used to develop the genome sequences of *B. rapa* [30], *B. oleracea* [31], *B. nigra* [32], *B. napus* [33] and *B. juncea* [32]. The genomes of all Brassica species underwent a lineage-specific whole-genome triplication, followed by diploidization that involved substantial genome reshuffling and gene losses [32]. Another application of NGS is gene mapping through mapping by sequencing, which has been used to map important genes in the genomes of organisms such as *Arabidopsis thaliana* [34–36] and pok choy [19]. In addition, the CR gene *Rcr7* was successfully mapped in cabbage through analysis of percentage of polymorphic variants (PPV) based on bulked segregant RNA sequencing (BSR-Seq) [25].

A previous study identified *B. nigra* accession PI 219576 as highly resistant to clubroot [7]. The objectives of the current study were to: i) identify the resistance gene in PI 219576; ii) map the resistance gene using genome sequencing information from the A-genome of *B. rapa* and B-genome of *B. nigra*; iii) develop SNP markers tightly linked to the resistance gene; and iv) identify the most probable candidates for *Rcr6*.

## Results

### Inheritance of CR in line PI 219576

**Fig. 1:**
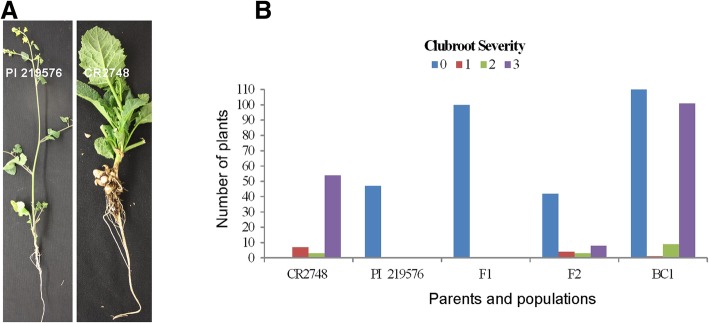
Evaluation of clubroot disease reaction to inoculation with pathotype 3 under controlled conditions: (**a**). phenotypes of resistant line PI 2195760 and susceptible (CR2748) line; (**b**). distribution of phenotypes in *Brassica nigra* parental lines (PI 219576 and CR2748) and the F_1_, F_2_ and BC_1_ populations

The parental line PI 219576 was highly resistant to pathotype 3 and inoculation produced no clubroot symptoms. Line CR2748 was highly susceptible with large galls (Fig. 1a). The F_1_ generation derived from the cross of CR2748 with PI 219576 was highly resistance to pathotype 3 (Fig. 1b), indicating that PI 219576 was likely a homozygous resistant line and the resistance was dominant. The F_2_ plants from the cross showed a 3:1 segregation ratio for R and S (Fig. 1b, Table 1). Evaluation of BC_1_ plants from PI 219576 showed a 1:1 segregation ratio (Fig. 1b, Table 1), indicating that CR in PI 219576 is controlled by a single dominant gene designated as *Rcr6* (*Resistance to* *club* *root* *6*).

**Table 1:** Genetic analysis of resistance to clubroot in the segregating populations derived from CR2748 (Susceptible, S) x PI 219576 (Resistant, R) with pathotype 3 of *Plasmodiophora brassicae*

		Phenotype				
Population	Total	R	S	Expected ratio (R:S)	χ^2^	*P-value*
BC_1_	234	123	111	1:1	0.62	0.43
F_2_	57	42	15	3:1	0.05	0.82

### Assembly of RNA-seq short reads into the reference genomes of *B. rapa*

The pooled sample assembly (PSA) of the three R bulks and three S bulks was carried out. A total of 32.0 million (M) sequences, 2062.2 Mb in length, with 8-fold coverage of the reference A-genome were assembled into *B. rapa* chromosomes from the pool of three R bulks, and 39.5 M sequences, 2523.4 Mb in length, with 10-fold coverage were assembled from the pool of three S bulks (Table 2). The sequence counts assembled into the genome for each chromosome were significantly correlated to chromosome length for the R bulks (r = 0.90, *P* = 0.003) and S bulks (r = 0.89, *P* = 0.0005).

**Table 2:** Short reads from the resistant (R) and susceptible (S) bulks were assembled into chromosomes of the *Brassica rapa* and *B. nigra* reference genomes

Chromosome number	Chromosome size (bases × 10^6^)	Number of sequences (× 10^6^)		Accumulated length of sequences (bases × 10^6^)	
		R	S	R	S
*B. rapa*					
A01	26.8	3.0	3.7	191.4	233.8
A02	27.0	2.8	3.4	176.4	215.3
A03	31.8	4.4	5.4	284.8	342.2
A04	19.3	2.6	3.3	170.4	210.7
A05	25.3	3.0	3.6	192.5	230.7
A06	25.2	3.4	4.4	219.2	285.0
A07	25.9	3.3	4.0	211.6	255.5
A08	20.8	3.0	3.6	192.9	234.6
A09	38.9	4.5	5.6	292.2	356.2
A10	16.4	2.0	2.5	130.8	159.4
Total	257.4	32.0	39.5	2062.2	2523.4
*B. nigra*					
B1	42.3	5.0	5.7	345.6	395.1
B2	52.7	5.3	6.2	362.6	426.7
B3	46.9	5.6	6.4	385.9	449.1
B4	43.4	4.8	5.6	334.6	393.3
B5	51.8	5.0	6.0	347.5	415.9
B6	36.7	5.9	6.9	407.7	481.9
B7	41.7	4.1	4.8	281.6	331.0
B8	54.2	5.4	6.3	375.8	439.9
Total	369.7	41.1	47.9	2841.3	3332.9

### Mapping of *Rcr6* with PPV against the A-genome

**Fig. 2:**
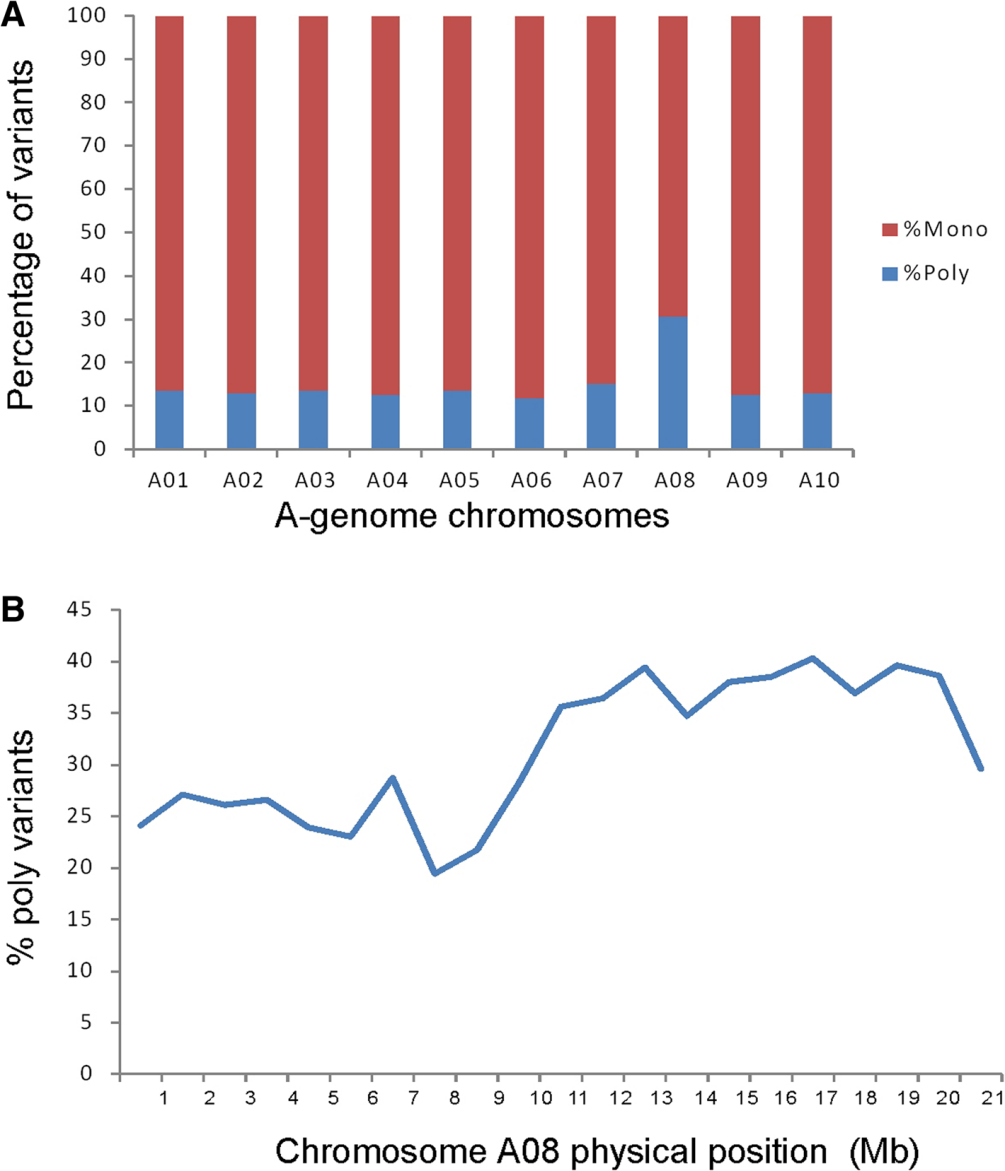
Analysis of BSR-Seq to map *Rcr6* based on the reference genome of *B. rapa*: (**a**). the percentage (%) of monomorphic and polymorphic variants on each chromosome; and (**b**). % polymorphic variants on chromosome A08

In total, 120.2 K polymorphic (poly) variants (SNPs and InDels) were identified when aligned with the A-genome. Variants in the *B. rapa* genome generally consisted of 85–88% monomorphic (mono) variants and 12–15% poly variants. The only exception was chromosome A08, which carried more ploy variants (31%) and fewer mono variants (69%) than the other chromosomes (Fig. 2a). This difference relative to A08 indicated that *Rcr6* may reside in a genomic region in the B-genome of *B. nigra* homologous to A-genome chromosome A08. The PPV on chromosome A08 was further analyzed. The highest PPV was located in the physical range from approximately 10–20 Mb (Fig. 2b), indicating that *Rcr6* is located in a genomic region homologous to 10–20 Mb of chromosome A08.

### Defining *Rcr6* location relative to the A-genome

**Fig. 3:**
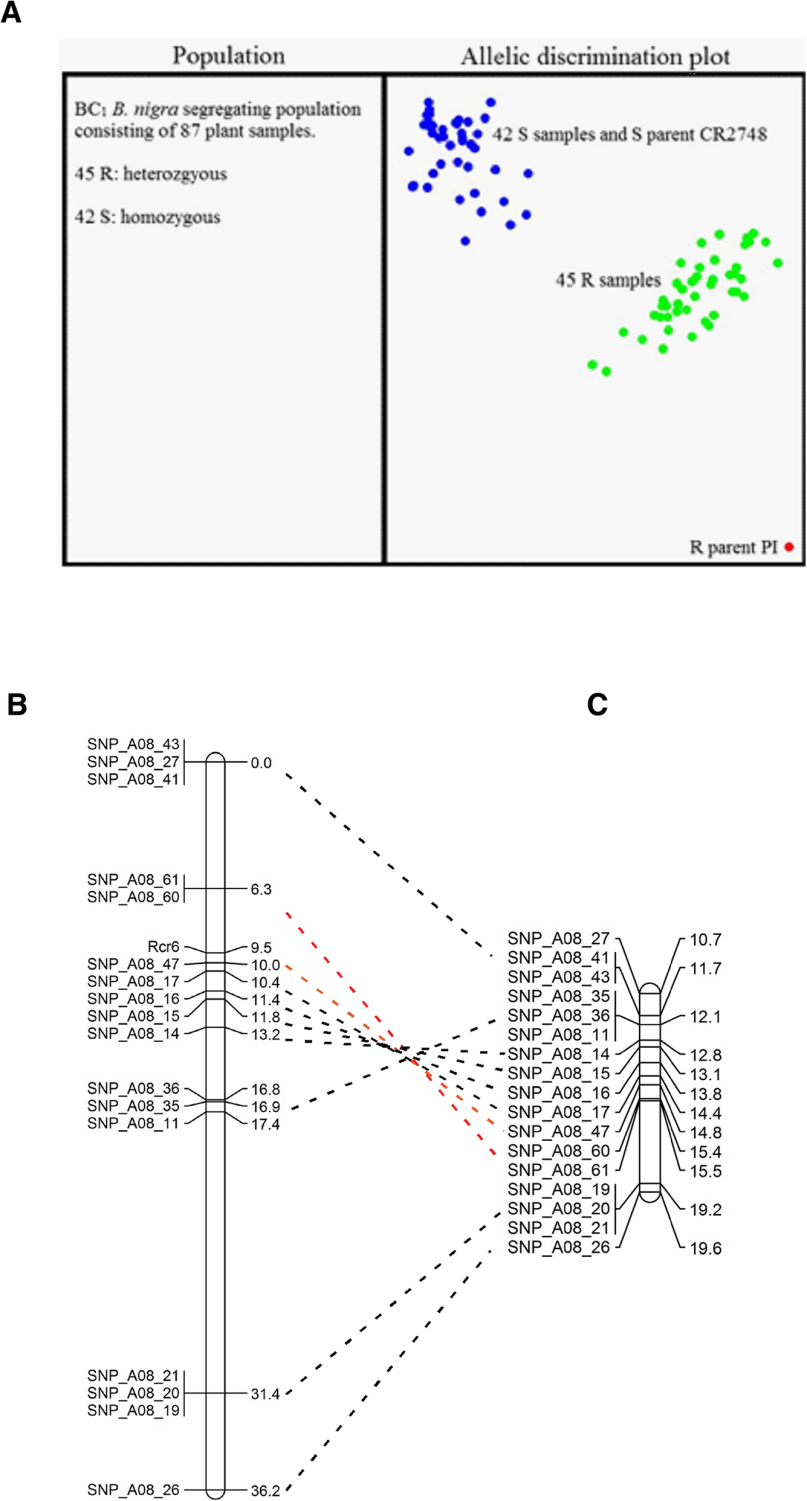
Genetic mapping of *Rcr6*: (**a**). genotyping of SNP markers using KASP. The R parent (homozygous resistant) segregated to the lower right quadrant, the S parent (homozygous recessive) and S individuals (homozygous recessive) from BC_1_ population formed a cluster in the upper left quadrant, and the R individuals (heterozygous resistant) from the BC_1_ population were positioned between the S cluster and R parent; (**b**). genetic map of the region in which the *Rcr6* gene is located (genetic distance on right); and (**c**). physical location of the *Rcr6* region (in bases, on right), with SNP markers connected with a broken line between the maps

The 221 plants in the BC_1_ population were assessed using KASP analysis of 17 poly SNP sites against chromosome A08; these sites spanned a distance of 10.7 Mb to 19.8 Mb (Fig. 3a). A linkage map of 36.2 cM was constructed (Fig. 3b), which confirmed that *Rcr6* lay in a region homologous to 10–20 Mb of chromosome A08. Homology between chromosome A08 of *B. rapa* in the region and the corresponding region in *B. nigra* genome was confirmed through genetic mapping. However, the DNA fragment from the markers SNP_A08_61 to SNP_A08_11 in the *Rcr6* region (Fig. 2c) was reversed comparing with the *B. rapa* chromosome (Fig. 3c). *Rcr6* was flanked by SNP_A08_60 & 61 and SNP_A08_47, in an interval of 3.7 cM. The flanked segment was homologous to the region between 15.4 Mb and 14.8 Mb of chromosome A08 (Fig. 3c), spanning about 0.6 Mb region. There were 190 genes annotated in this region of reference genome v1.5, with five genes (*Bra010552*, *Bra010588*, *Bra010589*, *Bra010590* and *Bra010663*) identified as encoding TNL class of proteins (Additional file 4: Table S1).

### Identification of the *Rcr6* region in the draft *B. nigra* genome

The short reads from the BSR-Seq project were assembled into Dr. Parkin’s draft *B. nigra* genome sequence after mapping of *Rcr6* with the *B. rapa* reference genome was completed. A total of 41.1 M sequences, 2841.3 Mb in length, with 8-fold coverage of the reference B-genome were assembled into *B. nigra* chromosomes from the pool of three R bulks; and 47.9 M sequences, 3332.9 Mb in length, with 9-fold coverage were assembled from the pool of three S bulks (Table 2). More sequences were aligned into chromosomes B3 and B6; fewer sequences were aligned into chromosomes B4 and B7 than in the shortest chromosome B6 (Table 2). However, the sequence counts assembled into the genome for each chromosome were not correlated to chromosome length for the R bulks (r = 0.07, *P* = 0.87) and S bulks (r = 0.11, *P* = 0.79).

**Fig. 4:**
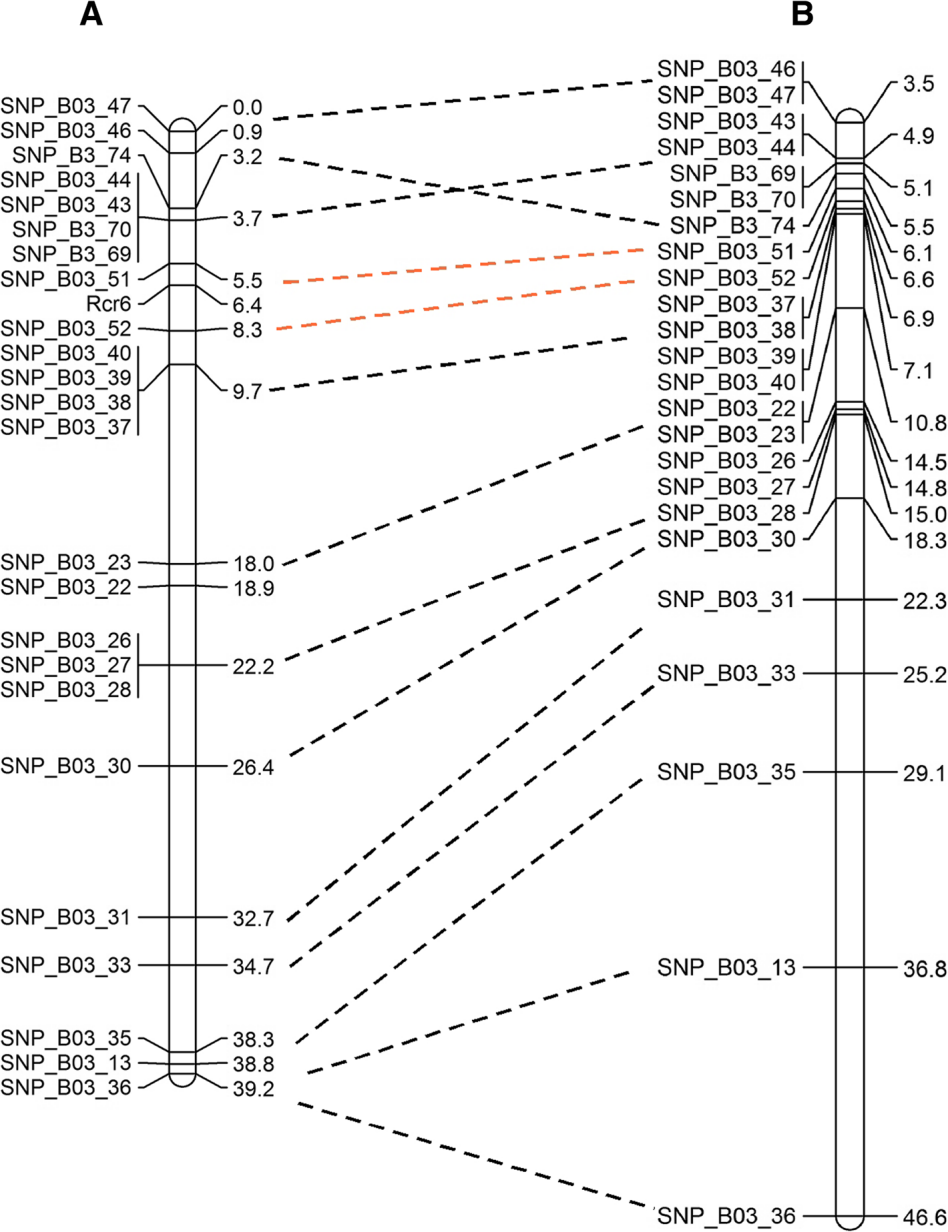
Mapping of *Rcr6* into chromosome B3 using the draft genome of *B. nigra* from Canada: (**a**). genetic map of the region in which the *Rcr6* gene was located (genetic distance on right); and (**b**). physical location of the *Rcr6* region in bases (on right) and genetic location, with each SNP marker connected between the two maps with a broken line

The variants identified across the *B. nigra* usually consisted of 63–67% mono variants and 33–37% poly variants, except for chromosome B3, which carried fewer mono variants (51%) and more poly variants (49%) than the other chromosomes (Additional file 1: Figure S1A). This indicated that *Rcr6* was located in *B. nigra* chromosome B3. Also, there was a peak in PPV in the 36–37 Mb region of B3 (Additional file 1: Figure S1B), but there were very few poly variants in the region (Additional file 1: Figure S1C). A SNP marker, SNP_B03_13, located in 36.8 Mb of chromosome B3 was genotyped using KASP. However, it was not closely associated with *Rcr6* (Fig. 4a), indicating that *Rcr6* was not in the region.

The BC_1_ population consisting of 221 plants was analyzed with 23 SNP markers on chromosome B3 and a linkage map covering 39.2 cM was constructed (Fig. 4a). *Rcr6* was flanked by SNP_B03_51 and SNP_B03_52, in an interval of 2.8 cM. The flanked segment is homologous to the region of 6.1–6.6 Mb on chromosome B3 (Fig. 4b), spanning about 0.5 Mb. A joint map was also constructed with SNP markers developed based on both chromosomes A08 and B3 (Additional file 2: Figure S2).

There were 109 genes predicted in the *Rcr6* target region of 6.1–6.6 Mb on chromosome B3. Analysis with Blast2Go indicated that only one gene (FGENESH_B3_72) encoded the TNL class of proteins (Additional file 4: Table S2). This *B. nigra* TNL gene is homologous to the *B. rapa* gene *Bra010663*, which is located from 15,082,118 to 15,087,789 bases of chromosome A08, based on a blast search at http://brassicadb.org/brad against the *B. rapa* reference genome.

### Determining the location of *Rcr6* in the published *B. nigra* genome

The short reads from the BSR-Seq project were also assembled into the published *B. nigra* reference genome v1.1 at http://brassicadb.org/brad. Analysis of PPV was also performed following the identification of genome wide DNA variants. The variants identified across the *B. nigra* genome usually comprised of 61–63% mono variants and 37–39% poly variants. Unexpectedly, chromosome B7 [32] carried fewer mono variants (49%) and more poly variants (51%) than the other chromosomes (Additional file 3: Figure S3A). This indicated that *Rcr6* was located in the *B. nigra* chromosome B7 (designated as B7_China in the current study). This result also indicated that chromosome B3 sequenced by the research group in Canada (designated as B3_Canada) is equivalent to B7_China. Pairwise chromosome alignments of B3_Canada with the reference genome of *B. nigra* confirmed that B3_Canada was the same chromosome as B7_China. However, the orientation is opposite (Additional file 3: Figure S3B).

Blast search in the *B. nigra* reference genome v1.1 was performed using the DNA fragment in the *Rcr6* target region defined by SNP_B03_51 and SNP_B03_52 at http://brassicadb.org/brad. This fragment hit B7_China with the highest score of 2.427e^+ 04^. The TNL gene of B3_Canada in the *Rcr6* interval homologous to the *B. rapa* TNL gene *Bra010663* hit the *B. nigra* TNL gene *BniB015819* in B7_China with a score of 729.0. This gene is located into 36,680,688 to 36,684,758 bases of B7_China.

## Discussion

*Brassica* species with the B-genome contain a large reservoir of genes conferring resistance to important diseases of canola such as clubroot and blackleg. Resistance to clubroot in *B. nigra* could be transferred into B-genome species such as *B. juncea* and *B. carinata* by interspecific hybridization or re-synthesis of these species. Due to differentiation among the *Brassica* genomes caused by genome rearrangements and gene deletions, the level of homology between B and A/C chromosomes is low [37], which makes introgression of genes from the B-genome into the A or C-genomes of canola (*B. napus*) difficult through conventional breeding. To facilitate the transfer of resistance available in the B-genome into canola, it is necessary to accurately map genes in *B. nigra*, so that molecular markers tightly linked to these resistance genes can be identified for use in marker-assisted selection (MAS). Alternatively, the genes could be isolated from *B. nigra* so that the genes could be delivered into canola through gene transformation.

When the project to map *Rcr6* was initiated, no reference genome for *B. nigra* was available. Therefore, *Rcr6* was initially mapped against a reference A-genome of *B. rapa* [30]. The genetic location of *Rcr6* was further defined using SNP markers identified though BSR-Seq based on the *B. rapa* reference genome. This demonstrated that mapping by sequencing was possible in a species using the genome sequence from a close relative if its own genome sequence was not available. In the current study, *Rcr6* was mapped into chromosome A08 using the reference genome from *B. rapa*.

A draft genome of *B. nigra* sequenced in Canada subsequently became available. *Rcr6* was then mapped into *B. nigra* chromosome B3 (designated here as B3_Canada), which indicated that the target region of *Rcr6* in B3_Canada of *B. nigra* was homologous to the corresponding region in chromosome A08 of *B. rapa*. *Rcr6* was also mapped using a published *B. nigra* sequence from http://brassicadb.org/brad in China [32] into chromosome B7_China. The Canadian group used a nomenclature published by Lagercrantz and Lydiate [38], while the Chinese group accepted a proposal for nomenclature described by Panjabi et al. [39]. Therefore, it is not surprising that *Rcr6* was mapped into different chromosomes using these two separately derived sources of the *B. nigra* genome.

In a previous study, RNA-Seq and block homoeology analysis had identified synteny between the A and B-genomes in *B. juncea* [40]. Based on the current study, we believe that *Rcr6* could possibly integrate into A08 of *B. napus* in inter-specific crosses between *B. napus* and *B. nigra*, but this speculation has not yet been confirmed. Despite the challenges involved in transferring agronomically important traits from the B-genome species into *B. napus*, a blackleg resistance gene from the B-genome of *B. juncea*, *Rlm6*, has been successfully introgressed into *B. napus* [2].

Substantial effort has been made to identify genes or QTLs for CR in *Brassica* species containing A- or C-genome. In contrast, *Rcr6* is the first CR gene to be finely mapped in a B-genome species. It was located on chromosome B3_Canada, homologous to 14.8–15.4 Mb of Mb of chromosome A08. Five genes (*Bra010552, Bra010588, Bra010589, Bra010590* and *Bra010663*) were identified as encoding the TNL-class of proteins in this interval. The CR gene *Crr1* has previously been mapped into chromosome A08 [8] and cloned [20]. It is highly homologous to gene *Bra020861*, which located in the 10.8 Mb region of chromosome A08. In addition, a strong QTL, *Rcr9,* was also mapped into chromosome A08 [24]. The nearest SNP to *Rcr9* was A08_10272562. The current study indicated that *Rcr6* was not located on the corresponding genomic region of *Rcr9* or *Crr1* on chromosome A08. In the current study, the only TNL gene identified in the *Rcr6* interval is FGENESH_B3_72 (Additional file 4: Table S2), which is highly homologous to the *B. rapa* TNL gene *Bra010663* and the *B. nigra* TNL gene *BniB015819*. The availability of closely linked SNP markers will facilitate molecular cloning of *Rcr6* from the donor line. Although the cloned CR genes encode the TNL-class of proteins [20–22], it is possible that clubroot resistance is encoded by other classes of disease resistance genes. However, further clarification of the relationship between classes of disease resistance proteins and *Rcr6* was beyond the scope of the present study.

Breeding for CR can be severely constrained when the pathotype of interest does not occur near the site of established breeding institutions [19], so the use of molecular markers in marker-assisted selection can be extremely important. More than 10 robust SNP markers developed in this study were shown to be tightly linked to *Rcr6* using KASP. These markers could facilitate introgression of *Rcr6* into canola.

Until recently, only five pathotypes of *P. brassicae* (2, 3, 5, 6 and 8, based on Williams’ differentials) had been identified in Canada and pathotype 3 was the most prevalent pathotype on canola. Line PI 219576 of *B. nigra,* which was the resistant parent in the cross examined in the current study, was highly resistant to all of these pathotypes [7]. Recently, 12 new pathotypes of *P. brassicae* virulent on canola were identified using a new Canadian Clubroot Differential set [41]. PI 219576 also showed resistance to all of these new pathotypes (data not shown). *Rcr6* from PI 219576 was identified through testing for disease reaction to pathotype 3 only, so studies to confirm that *Rcr6* confers resistance to other pathotypes are needed.

RNA-Seq produces a large amount of short DNA sequence reads from random places in the transcriptome. The RNA-Seq data generated from the R and S bulks in the BC_1_ population was aligned into the A-genome of *B. rapa* and B-genome of *B. nigra*. This study characterized genome-wide variants in the *B. nigra* population that carried *Rcr6* and demonstrated that the sequence counts assembled into the A-genome of *B. rapa* for each chromosome were correlated to chromosome length from the R and S bulks, which was consistent with a previous study [19]. However, there was no correlation between assembled sequence lengths and the B-genome of *B. nigra*, which was not anticipated and requires additional study.

A previous study identified a high proportion of PPV on chromosome A03 of *B. rapa* adjacent to the CR gene *Rcr1* [19] and *Rcr7* was mapped into chromosome C7 of *B. oleracea* through identification of PPV [25]. In the current study, *Rcr6* was mapped into 10–20 Mb of A08 of *B. rapa* and *B. nigra* B3_Canada. However, the peak region based on PPV on B3 was not closely associated with *Rcr6*. One reason for this could be the low number of variants identified in the region due to a relatively low depth of RNA-Seq reads (Additional file 1: Figure S1). Greater depth of sequencing for genetic mapping via identification of PPV is recommended for future research.

## Conclusion

We aimed to identify novel CR genes from *B. nigra* that can be used in canola and mustard crops in the Canadian prairies. *Rcr6* was the first gene for resistance to clubroot identified and mapped in the B-genome of *Brassica* species. The sequencing information from *B. rapa* and *B. nigra* was used for BRS-Seq to map the gene. It resides in the genomic region of *B. nigra* homologous to chromosome A08 of *B. rapa*. Based on this funding, we believe that it could possibly integrate into A08 of canola crop *B. napus*. SNP markers tightly linked to the gene were developed, facilitating canola and mustard breeders for use of MAS in introgression of the CR gene into their cultivars. This gene was mapped into a small interval with one TNL gene (*BniB015819)* identified. This gene can be the candidate of *Rcr6* for gene cloning.

## Methods

### Plant materials

Nutrien Ag Solutions (201–407 Downey Rd., Saskatoon, SK, Canada) provided seed of the clubroot-resistant line PI 219576. The susceptible line CR2748 was provided by IPK (Leibniz Institute of Plant Genetics and Crop Plant Research, OT Gatersleben, Corrensstrasse 3, D-06466 Seeland, Germany). The resistant line (male) was crossed with the susceptible line (female) to produce the F_1_. Self-pollination of F_1_ plants produced the F_2_. A resistant F_1_ plant (male) was backcrossed with the susceptible line (female) to produce a BC_1_ population.

### Assessing clubroot resistance

Seed was planted in Sunshine #3 soil-less planting mix (SunGro Horticulture, Vancouver, BC) in tall, narrow plastic pots (5-cm diameter, 20-cm height, Steuwe & Sons, Corvalis, OR). The soil mix was treated with 1% (w/v) 16–8-12 (N-P-K) control-released fertilizer. Plants were maintained in a greenhouse (~ 22°/18 °C, day/night) with a 14-h photoperiod (230 μmol/m^2^/s at the canopy level). After inoculation with *P. brassicae*, plants were transferred to a growth room at 23°/20 °C and 14-h photoperiod (512 μmol/m^2^/s).

Pathotype 3 of *P. brassicae* (Williams’ differential system), which was originated from a canola field infected with clubroot in Alberta Canada, was used for inoculation throughout the study. The inoculum was prepared as a spore suspension with the concentration adjusted to 1 × 10^7^ resting spores mL^− 1^. For inoculation, 5 mL of spore suspension was applied adjacent to the seed, resulting in about 1 ×  10^6^ spores g^− 1^ growth medium. Inoculated seedlings in the growth room were maintained at a high soil moisture level for 2 weeks by retaining an excessive amount of water in the tray under the pots.

At 5 weeks post-inoculation, clubroot severity was assessed using a 0–3 scale where 0 = no clubbing; 1 = a few small clubs; 2 = moderate clubs; and 3 = severe clubs [42]; a rating of 0 was considered resistant (R) and 1–3 were susceptible (S). The F_2_ populations were analyzed to confirm the expected genetic analysis ratio of 3R: 1S and the BC_1_ populations (expected 1R: 1S ratio) were used for genetic mapping. The goodness of fit for the segregation was analyzed using a Chi-square (*X*^2^) Test [43].

### DNA extraction

DNA was extracted from young leaves using the CTAB method [44] with the following modifications: freeze-dried leaf samples were incubated with extraction buffer (2% CTAB; pH 8.0) at 65 °C, followed by extraction in chloroform-isoamylalcohol (24:1, v/v) and alcohol precipitation. RNA was eliminated by adding 1/10 volume of 10 mg/mL RNase A. The DNA concentration was estimated using a NanoDrop ND-2000c spectrophotometer (Thermo Scientific, Wilmington, DE).

### RNA-Seq

The BC_1_ population was used for RNA-Seq analysis. At 15 days post-inoculation, leaf tissues from 30 R plants were combined to form an R bulk, and tissues from 30 S plants were combined to form an S bulk. Together, the two bulks comprised one biological replicate. There were three replicates, with a total of 90 R and 90 S plants assessed. Total RNA from each bulk was extracted using the RNeasy Plant Mini Kit (Qiagen; Toronto, ON) with on-column deoxyribonuclease (DNAse) digestion using a Qiagen RNase-Free DNase Set, following the manufacturer’s instruction. RNA concentration and purity were checked using a NanoDrop ND-2000c spectrophotometer. The Experion RNA StdSens analysis kit (Bio-Rad Laboratories, Inc., Montreal, QC) was used for RNA quality analysis, with the Experion automated electrophoresis system. RNA quality was analyzed to ensure that the RNA integrity number (RIN) was > 8 for each sample.

cDNA libraries were prepared following the TruSeq RNA Sample Preparation v2 Guide (Illumina; San Diego, CA). The NanoDrop ND-2000c spectrophotometer was used to check cDNA concentrations and purity. Quality control and qPCR analysis were carried out to validate the cDNA libraries. The Experion DNA 1 K Analysis Kit (Bio-Rad Laboratories, Inc.) was used to confirm the size and purity of the cDNA libraries, based on a band at approximately 260 bps. The KAPA Library Quantification Kit v4.11 was used to perform qPCR of cDNA libraries.

RNA-Seq was carried out on samples from each inoculated R and S bulk using the Illumina MiSeq platform at the University of Saskatchewan (Saskatoon, SK, Canada).

### Sequence alignment, SNP discovery and mapping of the causal gene

No DNA sequence of the B-genome of *B. nigra* was available when mapping of the CR gene in *B. nigra* resistant line PI 219576 was initiated. Therefore, high-quality reads from BSR-Seq were aligned to the A-genome of *B. rapa* ssp. *pekinesis* (Chinese cabbage) cv. Chiifu genome v1.5 (http://brassicadb.org/brad) following PSA method described by Yu et al. [19] using SeqMan NGen 12 software (DNASTAR, Madison, WI). The reference A-genome consists of 10 chromosomes, with total combined length of about 257 million bases (Mb). Subsequently, a draft B-genome of *B. nigra* (developed by Dr. I. Parkin) became available for this study. The total length of the eight chromosomes in the draft genome was about 370 Mb. The short reads from the BSR-Seq project were aligned to the chromosomes of the draft genome. SNP discovery for marker development was carried out using SeqMan Pro 12 software (DNASTAR). The SNP discovery parameter was set at default, with Q call ≥15 and depth ≥ 5. Mapping of the causal gene was performed using PPV [25].

### Gene prediction and annotation

Gene prediction from the target region of the draft B-genome was performed at http://linux1.softberry.com/berry.phtml?topic=fgenesh&group=programs&subgroup=gfind using “Dicot, Arabidopsis (generic)” as the organism-specific finding parameter. Gene annotation for DNA fragments in the target region was analyzed with Blast2GO [45].

### SNP genotyping and linkage analysis

Selected SNPs identified through BSR-Seq were confirmed using the Kompetitive Allele Specific PCR (KASP) method (http://www.lgcgroup.com/), following the manufacturer’s instruction. PCR reactions were performed using a StepOne Plus Real Time PCR System (Applied Biosystem, Mississauga, ON, Canada). Genetic linkage maps were constructed by JoinMap 4.1 software, with distance measured in centimorgans (cM).

### Locating the causal gene in a published *B. nigra* genome

A *B. nigra* reference genome (v1.1) from China [32] became available at http://brassicadb.org/brad in 2017. Since this version had already been published and so was publically available, this was the version that was selected for the final version of alignment and analysis. The short reads from the BRS-Seq project were aligned to *B. nigra* v1.1 using SeqMan NGen 12 to identify the corresponding chromosome related to the causal gene. Additional confirmation was provided from pairwise chromosome alignments using two methods; i) the Blastn command line tool and the top hits with ≥98% to identity the query sequence in the reference genome, and ii) use LASTZ version 1.04.00 [46] to carry out pairwise chromosome alignments. Results were visualized using the Artemis comparison tool ACT [47]. The target region for the causal gene was searched at http://brassicadb.org/brad using the Blast tool.

## Additional files

Additional file 1: **Figure S1.** Mapping *Rcr6* based on BSR-Seg using a draft genome of *B. nigra* from Canada: A. The percentage of monomorphic and polymorphic variants on each chromosome; B. distribution of the percentage of polymorphic variants on chromosome B3; and C. distribution of polymorphic variants on chromosome B3. (JPG 434 kb)

Additional file 2: **Figure S2.** The genetic map of *Rcr6* with SNP markers identified through BSR-Seq from both chromosomes A08 and B3. (JPG 493 kb)

Additional file 3: **Figure S3.** Mapping of *Rcr6* into chromosome B7 using the published B-genome of *B. nigra* from China: A. the percentage (%) of monomorphic and polymorphic variants on each chromosome; and B. comparison of B3_Canada and B7_China. The dot plot was created using R (https://cran.r-project.org/). (JPG 1126 kb)

Additional file 4: **Table S1.** Blast2GO result in the Rcr6 target region of chromsome A08. **Table S2.** Blast2GO result in the Rcr6 target region of chromsome B3. (XLSX 59 kb)

## Data Availability

The datasets used and/or analyzed during the current study available from the corresponding author on reasonable request.

## Abbreviations

BSR-Seq: Bulked segregant RNA sequencing
CR: Clubroot resistance
MAS: Marker-assisted selection
PPV: Percentage of polymorphic variants
PSA: Pooled sample assembly
R: Resistant
*Rcr6*: Resistance to club root 6
S: Susceptible
SNP: Single-nucleotide polymorphism
TNL: TIR-NBS-LRR, toll-interleukin-1 receptor / nucleotide-binding site / leucine-rich-repeat
