# Supplementary figures and images for: Clubroot resistance gene Rcr6 in Brassica nigra resides in a genomic region homologous to chromosome A08 in B. rapa
Source: BMC Plant Biol. 2019 May 29;19:224. doi: 10.1186/s12870-019-1844-5 (PMC6542104; doi:10.1186/s12870-019-1844-5)

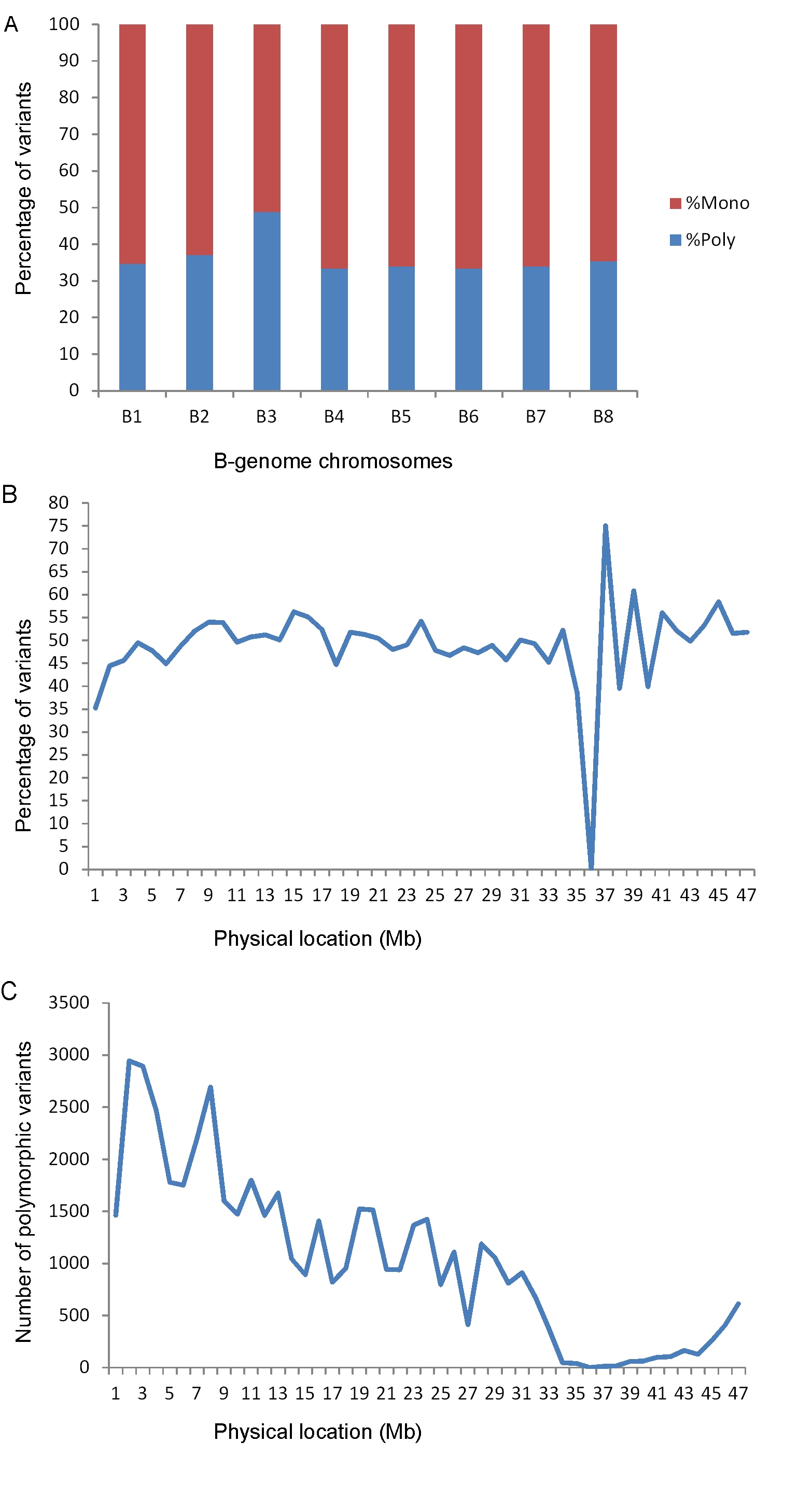

Supplement: Supplementary file 1 — Figure S1. Mapping Rcr6 based on BSR-Seg using a draft genome of B. nigra from Canada: A. The percentage of monomorphic and polymorphic variants on each chromosome; B. distribution of the percentage of polymorphic variants on chromosome B3; and C. distribution of polymorphic variants on chromosome B3. (JPG 434 kb) [file 12870_2019_1844_MOESM1_ESM.jpg]

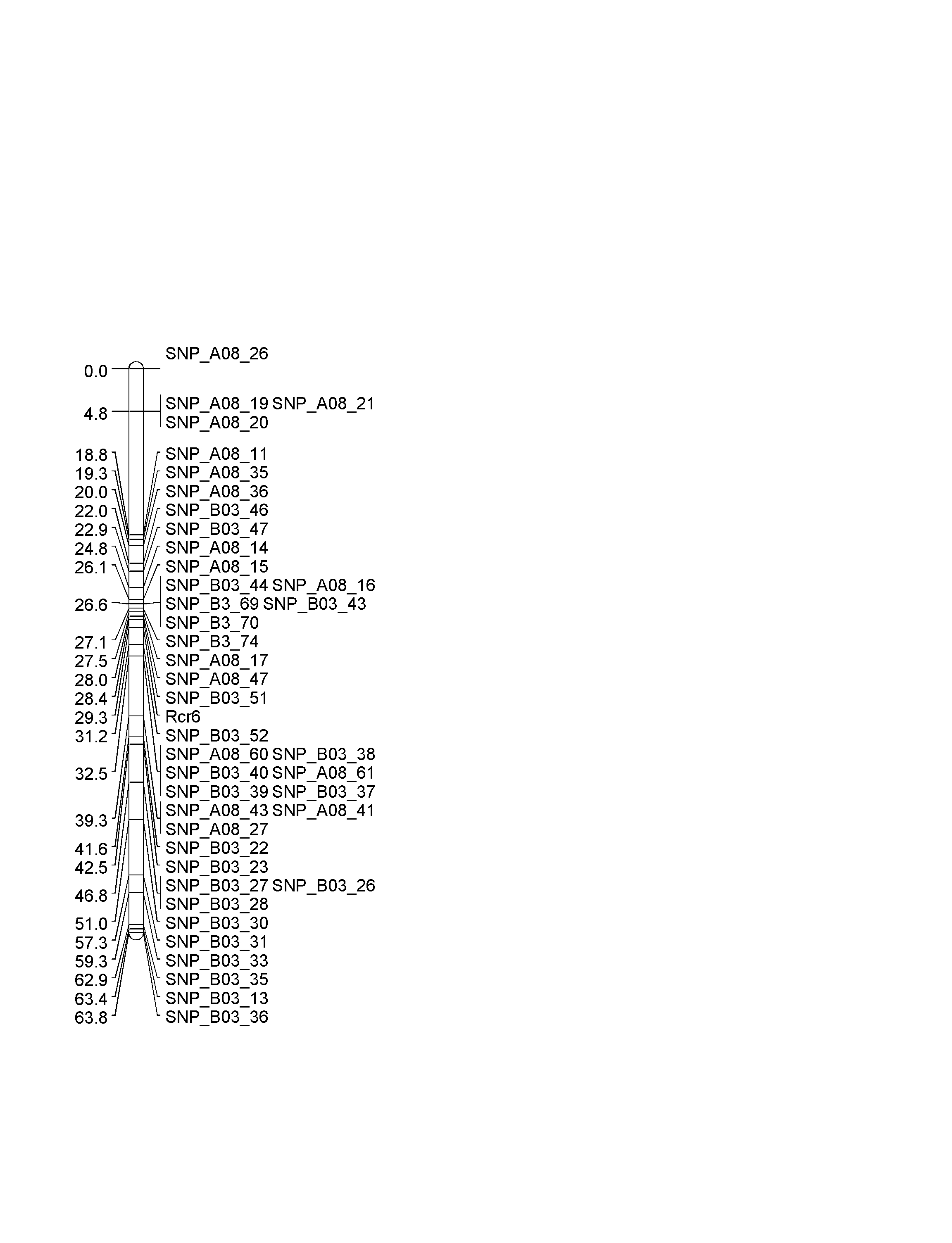

Supplement: Supplementary file 2 — Figure S2. The genetic map of Rcr6 with SNP markers identified through BSR-Seq from both chromosomes A08 and B3. (JPG 493 kb) [file 12870_2019_1844_MOESM2_ESM.jpg]

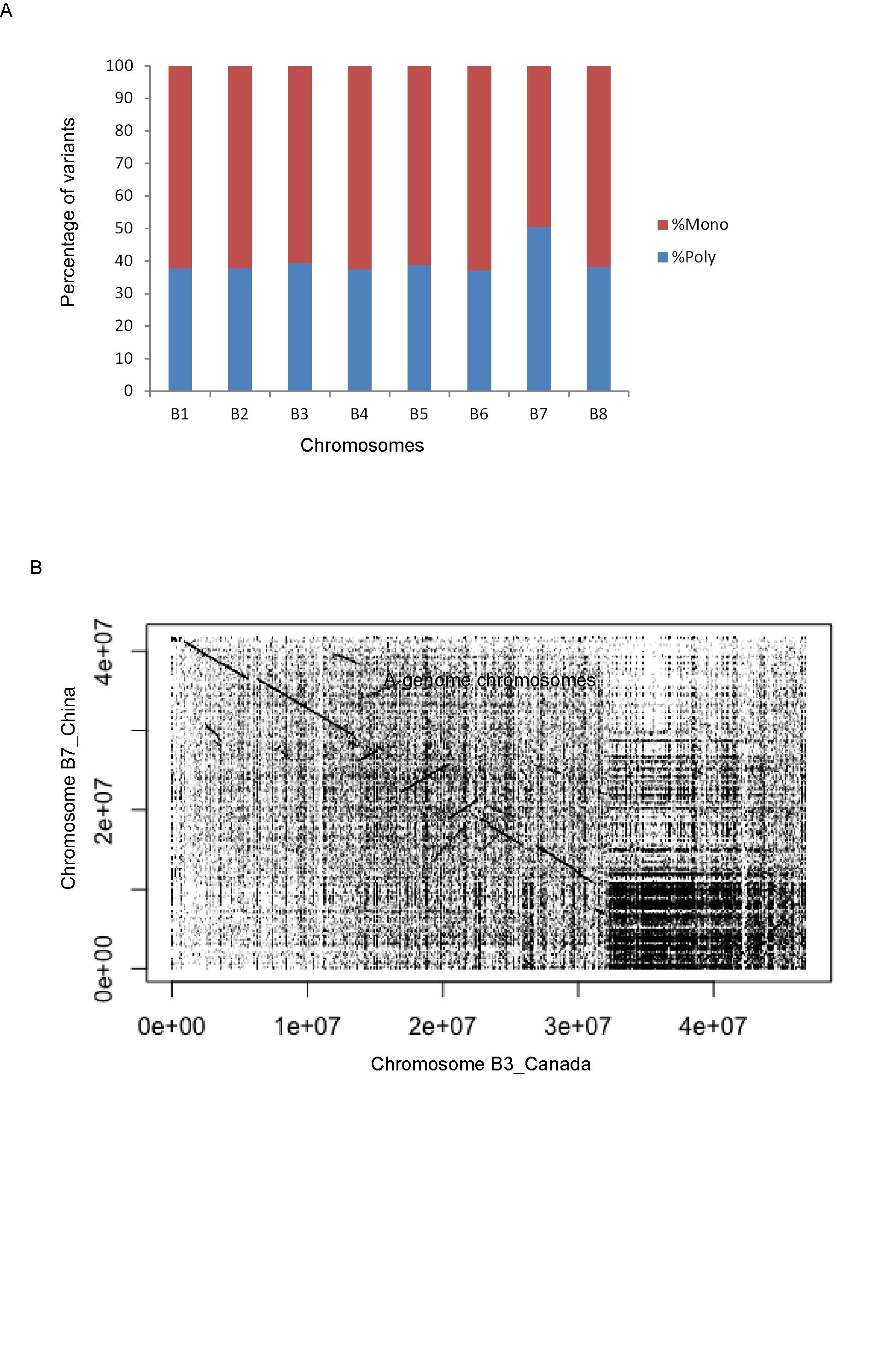

Supplement: Supplementary file 3 — Figure S3. Mapping of Rcr6 into chromosome B7 using the published B-genome of B. nigra from China: A. the percentage (%) of monomorphic and polymorphic variants on each chromosome; and B. comparison of B3_Canada and B7_China. The dot plot was created using R (https://cran.r-project.org/). (JPG 1126 kb) [file 12870_2019_1844_MOESM3_ESM.jpg]
